# Making Things Right: Nurses' Experiences with Workplace Bullying—A Grounded Theory

**DOI:** 10.1155/2012/243210

**Published:** 2012-04-08

**Authors:** Donna A. Gaffney, Rosanna F. DeMarco, Anne Hofmeyer, Judith A. Vessey, Wendy C. Budin

**Affiliations:** ^1^Columbia Center for New Media Teaching and Learning, Columbia University, 2970 Broadway, MC 4122, 603 Lewisohn Hall, New York, NY 10027, USA; ^2^William F. Connell School of Nursing, Boston College, 140 Commonwealth Avenue, Chestnut Hill, MA 02467, USA; ^3^School of Nursing and Midwifery, University of South Australia, City East Campus, GPO Box 2471, Centenary Building, Level 6, Room 54, Adelaide, SA 5000, Australia; ^4^NYU Langone Medical Center and NYU College of Nursing, 560 First Avenue, TCH-183, New York, NY 10016, USA

## Abstract

While bullying in the healthcare workplace has been recognized internationally, there is still a culture of silence in many institutions in the United States, perpetuating underreporting and insufficient and unproven interventions. The deliberate, repetitive, and aggressive behaviors of bullying can cause psychological and/or physical harm among professionals, disrupt nursing care, and threaten patient safety and quality outcomes. Much of the literature focuses on categories of bullying behaviors and nurse responses. This qualitative study reports on the experiences of nurses confronting workplace bullying. We collected data from the narratives of 99 nurses who completed an open-ended question embedded in an online survey in 2007. A constructivist grounded theory approach was used to analyze the data and shape a theory of how nurses *make things right* when confronted with bullying. In a four-step process, nurses place bullying in context, assess the situation, take action, and judge the outcomes of their actions. While many nurses do engage in a number of effective yet untested strategies, two additional concerns remain: inadequate support among nursing colleagues and silence and inaction by nurse administrators. Qualitative inquiry has the potential to guide researchers to a greater understanding of the complexities of bullying in the workplace.

## 1. Introduction

The situations are subtle and can range from sarcastic comments to being set up with the wrong patient chart… these sorts of things undermine your work day… erode your sense of comfort and security that you need to do your job in a professional manner (Nurse 41, 2007). 

The consequences of workplace bullying are as evident today as they were one hundred years ago. In 1909 Leon Harris condemned the treatment of nurses by their managers in an article published in *The New York Times*. Dr. Harris, citing multiple examples of workplace mistreatment, emphasized how “head nurses abuse their position of power” [1]. A century later the workplace has changed for the better in many parts of the world [2]. Yet, in spite of such advances, nurses still experience bullying in the workplace.

As the toll of workplace bullying has become more widely known in all work settings, research has dramatically increased. Many North American studies focus on behavioral categories, causes, and typologies of individual responses [3]. There is limited information on how nurses experience and resolve workplace bullying. While bullying in the healthcare setting has been internationally recognized and researched [4, 5], many institutions minimize its impact or deny its existence, creating a culture of silence that impedes solutions to this problem [6–8]. While most individuals consider bullying to be a highly overt behavior, it is usually an insidious form of workplace aggression causing professionals to distance from each other fearing social exclusion or becoming the target of abuse. The consequences of bullying include compromised performance, job dissatisfaction, increased absenteeism, and staff turnover [4, 5, 9]. Newly graduated nurses are at significant risk as evidenced by higher resignation rates during the first year of practice [10–12].

Workplace bullying has also been acknowledged as a threat to patient outcomes and the delivery of quality of patient care, as well as the erosion of personal health and professional wellbeing [9, 13, 14]. Excellence in patient care flourishes in an environment built on open communication and respectful professional relationships. An environment that condones bullying perpetrates destruction of professional communication.

Bullying goes by many names: workplace aggression, indirection aggression, social or relational aggression, horizontal (lateral) violence, and workplace violence. It has become so popularized in the press; bullying is often, mistakenly, used as an overarching concept. There is a tendency to use many of these terms interchangeably [8]. Bullying is different from horizontal violence in that a real or perceived power differential between the instigator and recipient must be present [15]. Some of the most recent literature suggests that all of these behaviors exist on a conceptual continuum of workplace victimization [3].

Authors from different disciplines have contributed to the proliferation of constructs that label hostile workplace behaviors [16, 17]. Bullying is a contested concept. Instead of agreement on a universal concept and definition, authors have added to the challenges of building a relevant literature base and conceptual framework. Europeans have led research and policy advances in the field for over three decades. Swedish psychologist Heinz Leymann was the first to study workplace bullying in a systematic way, finding that these negative actions occurred frequently and over time, causing physical, emotional, and social consequences [18]. Others define bullying as repeated, offensive, abusive, intimidating, or insulting behaviors, abuse of power, or unfair sanctions [12, 19]. These negative social acts, not only occur repeatedly and regularly over time but also escalate and occur between individuals who have different positions of power [15]. Saunders et al. [20] suggest that the characteristics of negative actions and harm are the essential elements of bullying.

Scholars have underscored the importance of the durational characteristics of bullying [15, 21]. The dual dimensions of frequency and duration of bullying actions distinguish it from day-to-day social stresses or poor management styles in the workplace. Keashly and Jagatic propose that repetition is a principal characteristic of bullying; yet few studies have explored the repetitive experience of bullying, either by frequency, duration, patterns, or escalation [17].

The relationship between negative psychological consequences and workplace bullying is well established [3]. In addition to the emotional and cognitive effects, there are physiological consequences [13]. Recipients of bullying feel humiliated, vulnerable, or threatened, thus creating stress and undermining their self-confidence [12, 19].

Prior to the last ten years, the nursing literature leaned towards anecdotal reports or articles suggesting practical solutions for dealing with workplace bullying [22]. Outside of nursing, research has been conducted using quantitative studies, primarily prevalence surveys. This complex phenomenon may require qualitative approaches for a fuller explication [23]. Such methods will allow us to unearth the interaction of individual, organizational, and cultural factors that enable, trigger, and reward bullying [6]. To this end, workplace bullying must be seen as a social process, “in which the impact on the person experiencing it is of primary importance” [24].

This qualitative study was part of a larger survey whose purpose was to validate the occurrence and patterns of bullying among nurses in the US [8]. The second-level qualitative analysis of the nurses' narratives describing their bullying experiences in health care settings is presented in this paper. We specifically sought to understand how nurses encounter bullying in the workplace and the strategies they use to protect themselves and their patients.

## 2. Methods

The approach adopted for this qualitative study was based on constructivist grounded theory methods [25, 26]. Charmaz contends that theory emerges not solely from the data but in concert with the individual experiences of the participants as well as values and experiences of the researcher [25, 26]. In a constructivist approach, the central question addresses how social reality is constructed. In addition, the researcher seeks to identify the elements of that reality [27]. To that end we were guided by both questions. First, we wanted to know *how* the social reality of bullying in the workplace came together for nurses and second *what* are the elements and organization of their reality. Working back and forth between these two questions throughout the analysis allowed us to make visible important aspects of the nurses' world and understand their experience of bullying. In a constructivist approach, Charmaz [25, 26] suggests that data and its analysis are social constructions leading us to ask what is the purpose of the narratives and how does the setting influence the phenomenon?

Complete objectivity is not possible by the researcher, “constructivist grounded theory assumes relativity, acknowledges standpoints and advocates reflexivity” [28]. Researchers bring their own values, experiences, and biases to the research process. Examining the relativity of perspectives, positions, practices, interactions, and the research situation is crucial to the process. With Charmaz's premise in mind, we acknowledge that the members of our research team come from different viewpoints to understand bullying in the nursing workplace. The diversity of our perspectives and experience are convergent, not biasing our approach. Our values and experiences complement one and other, allowing us to see the perspectives of the participants through different lenses. Our reaction to workplace bullying particularly in the health care setting and specifically among nurses was consistent. It was precisely our own workplace experiences and listening to the stories of nurses that brought us to formally investigate this problem.

Markham states, “an ethical researcher is a reflexive researcher who works from the heart” [29]. Being reflexive throughout the research process focused us on understanding how we contributed to the construction of meaning. Repeatedly, we stopped at critical junctures and explored why and how we came to an interpretation or a particular decision. This required a constant vigilance in rediscovering and sharing our own values, interests, politics, and even the influence we had on each other.

Because we conducted the study in a virtual setting, we thought a great deal about our respondents, who they are, and where they worked. Our participants existed both online and offline, and we reflected on their location within those worlds, a connected space of sorts [30]. We also understood the possibility that what happens in the online world is interwoven with the offline real world; there can be mutual shaping of the two [31]. Within the communication context, there is an interaction of the encounter and the virtual space [31].

Permission to conduct the study was obtained from the institutional review board (IRB) at a large university in the state of Massachusetts.

### 2.1. Data Collection and Analysis

An Internet web link to a 30-item anonymous e-survey was created [32] and appended to an article about workplace bullying in *Nursing Spectrum* a hard-copy and web-based, free, biweekly nursing magazine [33]. Consent to participate was affirmed by respondents completing the online survey and posting the optional narrative. The respondents were anonymous and not matched to the data of the larger quantitative study [8]. The optional question in the online survey was offered to participants as follows, “If you would like, in the space below please describe the bullying situation as you remember it. Please refrain from using any identifiable data (e.g., names, specific hospital, etc.).” The Internet web link was open for participant responses for a three-month period.

 A total of 99 narratives were submitted through the online survey and downloaded into Microsoft Word. Eleven responses were removed from analysis because the respondent offered commentary, broad generalizations, or opinions. Another six narratives were removed from analysis because they met the US Equal Employment Opportunity Commission definitions of harassment (sexual, disability, racial, or national origin) [34]. One narrative was removed because a nurse did not write it. A total of 81 narratives, ranging from five words to 780 words, were analyzed. Some narratives were very brief, “it is too painful to talk about,” while others wrote several hundred words describing who, what, when, where, how, and the consequences of working in a hostile environment.

Prior to open coding, we performed preliminary readings to capture the tone of each narrative and become attuned to the text, allowing us to gain a holistic understanding of the respondents' experiences before further analysis.

Charmaz's [26] approach to coding is multilayered. To optimize our sensitivity and carefully attend to the nurse's perspectives, we first coded narratives as a team and later, the first author led continued coding. We used the constant comparative method [35] to make comparisons at each level of our analysis looking for similarities and differences. We began with open coding (line by line) allowing us to look closely at the responses and reflect on the substance of the narratives. In some cases the nurses' words provided initial code names (in vivo codes). During the second phase we began focused coding by taking the most significant and frequently occurring earlier codes to sort through the data. The next step allowed us to identify linkages and connections. We developed categories by clustering similar codes, and from those categories we generated hypotheses about how the categories were related. We then moved to the discovery of a core social process [36]. We used theoretical coding to integrate the emerging theory. Theoretical coding allowed us to go beyond description and specify properties of and relationships between categories. We used Charmaz's analytic categories of agency, action, power, networks, and narrative and biography to further investigate the data at the stage of theoretical coding [26]. Throughout each phase of this process, we wrote memos, conferred with each other, and reached agreement on codes, categories, and concepts. We revisited the text of the nurses' responses throughout the analysis.

## 3. Findings

### 3.1. A Grounded Theory of Making Things Right

When the participants in this study confronted bullying, they expressed how their efforts were directed towards making the situation better for themselves, their colleagues, and on many occasions patients in their care. The discovery of the core category, *making things right*, and the four linked categories illuminate how the participants move through this central process.  Table 1 provides an overview of the categories and subcategories. These categories developed into a logical set of interrelationships and became integrated into steps. The first of these, *placing bullying events,* provides the contextual background for the core category. The three other categories are dependent on and linked to the core category: *assessing the situation*, *taking action,* and *judging outcomes*. Subcategories further described the characteristics of the four categories. Time, milieu, and interpersonal dynamics are critical dimensions of the above-mentioned categories.

### 3.2. The Core Category: Making Things Right

To illustrate the grounded theory of *making things right* we present here the narrative of Nurse 5 who talks about her own experience as a new nurse. For clarity, respondents' words were identified by the title of Nurse, followed by the case number, for example, Nurse 1, Nurse 2.

I was brand new and my preceptor for the shift was ill so I was assigned to precept with someone else… and if I did not do every little thing to her standard she stopped me and loudly announced to all “she did not do this or that” as if I were in a bad nurse spotlight!.. Her attack finished later that night by exclaiming I had done something without her there to watch and then claimed I rolled my eyes at her! She was menacingly close to my face and threatened me with the nurse manager. No one stood up for me… This nurse has repeatedly done this over the years and gets away with it. I recorded dates and events and brought them to my nurse manager(s), which resulted in my being blamed that I need to stand up for myself, confront her and she will then somehow respect me. I felt so alone. I was scared having never experienced this sort of thing before. Many of my coworkers never gave me a chance they played 6th grade girl mind games. I learned to ignore much…in the end I left that unit standing tall. I had regained my dignity because she did not destroy me and my coworkers were secretly glad.

As with many of our respondents, Nurse 5 describes how she was victimized. Aquino and Thau emphasize that this description is a necessary step in advancing the current state of workplace bullying research [3]. Nurse 5 deals with bullying situations by using a process of *making things right*. Beginning with *placing the bullying event *in context, she describes multiple bullying episodes and being in a “brand new” stage of her nursing career. Further description includes the very public nature of bullying as well as the more subtle actions of social aggression or “6th grade girl games.” Then *assessing the situation*, Nurse 5 not only recognizes the emotional and physical impact but also points out the shortcomings of the perpetrator. When *taking action* she identifies her strategies but also acknowledges inadequate support. Finally, when *judging outcomes* of the situation, she maintains her dignity and the respect of coworkers. Each of the four steps of the core category has subcategories that give depth to understanding how nurses make things right in the face of workplace bullying.

### 3.3. Category 1: Placing Bullying Events

Nurses who wrote about their experiences with bullying and hostility in the workplace began their narrative by defining their situation. Writers would often state when, where, and who was involved in the event. Some respondents identified the time as being “my first job” or a new clinical unit or being a student nurse. For others it was a detailed description of the words and actions taken by others in very specific situations. The context of the bullying event was situational or within an ongoing relationship. Aquino and Lamertz [37] report that victimization often emerges in the context of the dyadic relationship. Sometimes the nurses acknowledged that they were targets while others talked about witnessing hostilities and wrongdoings. *Placing* provides a framework of sorts, setting the stage for the next step of the process. Six subcategories emerged for *placing bullying events *in context: *being the newbie, bearing witness, in the bull's eye, nurse interrupted, odd nurse out, *and* being in the penalty box.*

**Being the Newbie**

Some respondents experienced bullying as new nurse. As noted above, Nurse 5 wrote about the searing memories of her first job, “the very first incident is burned in my heart and brain.” Another nurse (44) described her inadequate residency and the lack of support by other staff. Many times their descriptions seemed to have elements of hazing or being told that they were “ not good enough.”

**Bearing Witness**

The climate of bullying reveals itself to nurses as they “bear witness” to the mistreatment of others. Almost half of the respondents described numerous examples of the hostilities they saw. Their descriptions included the behaviors of the perpetrators as well as those who were targeted and bystanders. While these respondents were at a distance from the emotional fallout of the bully, many recognized the fear in their coworkers. Nurse 54 wrote about what she saw in the mistreatment of colleagues, “Many others were treated the same but they were “afraid” to speak up for fear they would lose their jobs and also afraid of the retaliation like I received.” The respondents also recognized patterns of resignations and firings, the worst possible outcomes. Nurse 6 describes the constant threat of termination on her unit, “I encountered two other nurses who had been fired for expressing the same concerns. On Friday of last week I was told of another firing in the same department when a nurse expressed the need for backup while doing conscious sedation and it was refused.” Respondents discovered breaches and other wrongdoings. They identified these mistakes and violations of policy and procedures as direct extensions of bullying with an impact on professional development and patient care.

**In the Bull's Eye**

Most of the respondents described in vivid detail how they were in the “bull's eye” and targeted for public censure and humiliation. They moved from observing at a distance to being at the center, the target of another's destructive behavior. Nurse 3 writes how she was under attack by another nurse, “She chose me to be her battering ram and on three separate occasions she unleashed her negativity on me. Swearing at me, criticizing what I was doing, demeaning me and just saying negative comments with others present.” Public humiliation and demeaning were a part of the process. One nurse (57) described the sting of public humiliation, “The administrator stated to me (in front of the other educator): “You should know when to listen to me and when not to.” I felt confused and humiliated. I truly thought I was doing a great job.” Respondents were also very sensitive to name-calling and in particular to being called “stupid” or incompetent. Power differentials and control are essential elements of workplace bullying [38], and abuses of authority further contribute to a state of dependency, as when workers describe infantilizing, diminishing actions, or being treated like a child [17, 39].

**Nurse Interrupted**

For most of the respondents, being a “nurse interrupted” was a daily experience. They witnessed or personally struggled with obstacles to patient care, assignment manipulation, having information withheld, being given incorrect or inadequate information, refusal of physical support, and being accused of incompetence. Skogstad et al. [40] propose that this behavior may also be an indication of changes in organizational structure and staffing patterns. Nurse 51, a recent transfer from a clinical unit to education, described how she and her students were blamed for errors and had critical patient information withheld.

Initially, the staff was not helpful to the students… as time progressed, the students and I began to be singled out and blamed for mistakes that the rest of the staff was making regularly. The staff nurses would avoid giving report to the students until I tracked them down and watched while they gave report.

Many respondents described how their patient assignments were manipulated. Nurse 54 simply said, “I would be given more patients than I could handle at one time. She would give me the most difficult assignments.” Nurse 41 struggled with inadequate information and unmanageable patient assignments and was also accused of poor nursing care.

The orienting nurse told me “You do not know home care, or hospice care because you come from a hospital, your thinking is all wrong. You will have to change the way you think.” She did not know I had previously worked in home care and hospice. She continued to tell me how my thinking and personality were wrong in the next few weeks… took credit for things I did for patients… told me I was on my own…“made” sure I did not even have the addresses or history on the patients I was to see, she had all of the information but would not share it. Then she told our new orienting supervisor that I was incompetent.

**Odd Nurse Out**

Social aggression took many forms for the nurses in this study. Negative behaviors harm another's self-esteem and/or social status and take the forms of verbal rejection or negative facial expressions [41–43]. Many of the respondents personally felt the “6th grade girl games” of collusion and exclusion. They talked about “feeling” that people were against them. Nurse 90 labeled the perpetrator of gossip and rumors, “She was a “tale carrier” from one site to another, often embellishing and focusing on weaknesses of staff and clients.” In the clinical setting collusion and exclusion, the hallmarks of social aggression look the same as in the schoolyard but with potentially more disastrous results for the professional nurse who is trying to fit in to the culture of the clinical unit. Nurse 80 stated the following.

The staff was threatening me that day, and all her friends ganged up against me when I reported it. Even though the person was transferred to another shift, her friends continued to give me a silent treatment causing me to be unhappy to come to work.

**In the Penalty Box**

Nurses felt they were being punished when they received sanctions, threats, or punishment. They wrote about being “demoted” (Nurse 55), “written up” (Nurse 61), or told to leave because nurses were “a dime a dozen” (Nurse 35). Nurse 71 described what led up to the restrictions placed on her practice.

I was made to work 7 days in a row. On three of those days I was the sole provider. On the 7th day I made multiple errors typing in data on the computer. I was pulled aside and told I made many mistakes and patient safety was in jeopardy. Since that time I have been told I may ONLY perform healthy physicals.

### 3.4. Category 2: Assessing the Situation

Once the respondents described and *placed* the bullying event they engaged in self-reflection, analyzing not only their reactions and roles but the environment as well, the subcategories of *reflecting on self* and *deconstructing the milieu *emerged. Their self-inventories included assessment of positive and negative emotional responses, feelings of powerlessness and frustration, and a shift in worldviews. Some nurses wrote about negative emotional responses. As with a number of other studies [3, 13], the participants admitted to feelings of stress, anxiety, anger, hopelessness, humiliation, fear, and even relief. Nurse 52 wrote how “This has been incredibly painful to me over the years.” Another respondent stated, “My self-esteem was so battered, I could not leave, sure that I was unemployable outside this venue” (Nurse 90). Nurse 2 addressed a loss of control and powerlessness, “When any kind of incident occurs we were accused of sloppy nursing care and incompetence without any inquiry of the facts surrounding the incident.”

Other respondents spoke of their competence and experience as a source of pride, even when others questioned their abilities. Nurse 90 recognized these qualities but still acknowledged the toll it took on her, “Now, clearly I have confidence in my abilities, judgment and assessment skills, and am respected and in demand for the same. Yet the damage remains.” Nurse 13 saw how her experience changed the way she viewed her profession, “The stress was awful. I eventually moved on to a better hospital/unit and excelled in my career. From then on I took new nurses and students under my wing and advocated for them.”

Many respondents described how the milieu, their own actions, or that of others contributed to the bullying situation. Nurse 95 deconstructed the milieu, “working the nights shift in ICU, being overwhelmed with work crises.” Nurse 63 admitted to her mistake, “My nurse manager exaggerated a medication error I made—very low class in the way she handled it. Yes, granted I did make the mistake but she did not have to call me in the middle of the shift to go off about it.” Some respondents saw the shortcomings of the perpetrator as a contributing factor, “My manager was underqualified and undertrained, yet had an inflated picture of her intelligence and worth. She thrived on power, enjoyed putting others in situations, where they would be uncomfortable or would fail (Nurse 90).” Once the situation was described and assessed, an approach to resolution of the situation followed what the nurse did to make things right.

### 3.5. Category 3: Taking Action

As the respondents identified the consequences of bullying and mistreatment in the workplace, many took action for themselves, their colleagues, and their patients. In many cases, the nurses were not victims or silent witnesses nor did negative emotions quash the proactive stance they took in the situations. More than half of the respondents detailed their actions. Using Lazarus and Folkman's [44] transaction model of stress, Aquino and Thau [3] classified strategies for dealing with workplace bullying as being problem focused or emotion focused. Problem-focused approaches centered on removing or dealing with the problem, either verbally or aggressively, escaping the situation or seeking support from others. The emotion-focused approach minimizes the negative emotional consequences by using internal coping strategies. In our study the nurses employed similar strategies when taking action. There were three distinct problem-focused subcategories of *taking action*: *giving and getting support, speaking up* (which included speaking out and whistle blowing), and *moving out of the toxic environment* by resigning or transferring from the work setting. Illustrations for the subcategories are presented below.

**Giving/Getting Support**

Of all the actions noted by the nurses, the least mentioned was giving/getting support. Very few respondents described getting support from colleagues, and one nurse wrote about how she gave support. Another nurse described the importance of support to her career satisfaction and healing in the aftermath of a bullying situation.

“My fellow staff nurses were wonderful and supportive. When I completed the orientation, I quit and went to work as a visiting nurse… I was treated like a valued member of the organization and did a lot of emotional healing.” (Nurse 66).

Many respondents offered detailed descriptions of *speaking up*, how they verbally or in writing voiced their concerns to nursing administration, physicians, preceptors, and others. Some would speak up to their peers, others would speak out and file formal complaints or reports, and a few reported wrongdoings to unions or other agencies. The narrative of this respondent (Nurse 39) weaves together the events, actions, and outcomes.

I was a 2-year nurse who went from the evening shift to the day shift. They (nurse managers) would assign me to the heavier patient workload and required me to do the narcotic count every time I came in. After being assigned to the stroke unit by the manager, both assistant nurse manager and the senior nurse stated that I should take 13 patients on the floor instead of the four in the stroke unit that day. I said “I will not, I have to stick to what I was assigned to me by the nursing manager (NM).” They yelled and said I was “unprofessional, would get in trouble, and should do what they say”—I kept my guard up, packed my things and told them that “if they did not want me to work I would go home” and left for the elevator. I told my supervisor in a calm manner. She told me to take the assigned stroke unit…. I got four patients and the silent treatment from the senior staff nurse and assistant manager (for 3 weeks)—but it was worth it!

Nurses also described resigning or transferring to a different unit or hospital, *moving out of the toxic environment. *However, it was clear from the nurses who decided to leave that this was a constructive move for them. The respondents used words like “poison” (Nurse 65), “dangerous” (Nurse 13), “laced with bullying” (Nurse 52), to describe their workplaces. They reframed their situation and eliminated the toxic environment, often finding satisfaction and reward in new settings.

### 3.6. Category 4: Judging Outcomes

The respondents discussed their evaluation and the consequences of their actions. Three subcategories emerged: constructive-positive outcomes, being ignored or no response and destructive-negative outcomes.

*Constructive-positive outcomes* were emphasized with words of joy and elation. Nurse 45 spoke up to a senior staff person who bullied her students. In doing so she vowed the bully would not berate or discipline her students. She described how she stood her ground. She smiled and “spoke firmly and turned on my heels and walked away, put my arm around my student and took her somewhere quiet and private where we could discuss the situation. ‘Nurse Ratchett' stood alone, in the middle of the hallway… I never heard another word from her. It felt great!!!”

**Being Ignored**

Many more respondents described how their appeals and actions were ignored. A number of key words were consistently used in those narratives: ignored, no action, did nothing, no follow through, or not backed. Frustration was apparent as one nurse (97) described multiple occasions of acting out behavior by another staff person, “all this was reported to administration but nothing was done for two years.”

**Destructive-Negative Outcomes**

Although many narratives mentioned fear of retribution or retaliation, some nurses described specific negative-destructive outcomes of their actions.

“I got” frequent write ups for anything possible to create a paper trail due to my union activity and standing up to defend our contract when they violated it. Eventually a patient family member threatened me and I verbally defended myself, they wrote me up and fired me for it (Nurse 64).

Another nurse (35) wrote about being threatened with termination.

I pointed out the danger of assigning one nurse to monitor nine infants, two months and younger with RSV, without monitors and on tank oxygen. They expected the same nurse to cover additional patient orders. My nurse manager told me if I did not like it, I could leave.

In addition to the measurable outcomes of being terminated or verbally threatened, humiliating nonverbal behaviors also occurred. Nurses talked about being given the “silent treatment” (39) or being labeled a “badmouth” (36). This outcome centered on a patient and family.

I had a dying patient in one room and a critical patient (involved in a procedure where I could not leave). The department head RN requested me to move the dying patient posthaste. I refused, as I knew death was imminent. She had the patient moved and the patient died in the elevator (Nurse 47).

## 4. Discussion

Nurses who try to *make things right* in the face of bullying or hostile work environments engage in a thoughtful process of analyzing their own roles as well as the actions of others and the resulting consequences. Nurses also see how workplace bullying diminishes the quality nursing care, placing patients at risk, whether it is from obstacles to performing nursing care, policy, or procedural violations.

The findings from this study illuminate the process of how 81 nurses responded to bullying in their workplace. Much of the nursing literature describes bullying events or characterizes those who bully but rarely move beyond the notion of labeling (from a myriad of concepts) or proposing theoretical solutions [22]. We learned from our respondents that they are not victims nor is bullying a singular event that overwhelms them. Nurses deal with workplace bullying on a day-to-day basis using a problem-oriented approach; with purpose, they move through a process of *making things right*. Because this process has multiple steps, there are multiple entry points for solutions. Nurses must be included in the discussion of effective strategies for each access point; that is, we can educate nurses that *placing *bullying in context has a number of different “faces” and that *taking action* can be giving and getting support as well as speaking up.

Many respondents in this study reported that they did not give or receive sufficient support. Support is crucial to *making things right *by allowing a professional to evolve from the bystander role of bearing witness to an upstander position of taking action. Samantha Power first used the word upstander to identify individuals who are willing to stand up and take action for themselves and others [45]. Rather than see nurses as victims, we must consider them proactive seekers of change and justice. Power's words are especially relevant for the nursing profession, “History has long been taught in terms of perpetrators and victims…but most of us live, actually in a different space, and that is the space not between perpetrators and victims but between bystander and, potentially, “upstander”.” [46]. For the most part nurses are not silent. However, their voices may be silenced before their message is heard.

 The findings in this study demonstrate that staff nurses frequently brought concerns of bullying, hostile behaviors, and threats to patient care to nurse managers when they could not effect change at the peer-to-peer level of interaction. The nurses described how they found their voices and took action despite stressful bullying experiences. They reported their perceptions to those in charge and asked for help. The nurses' narratives reveal that problems were deflected back with little or no assistance nor response from administration. Their self-advocating behaviors were undertaken with great risk, anxiety, and doubts as to whether they would be believed or seen as the problem. In some instances, the leaders and managers were silent or indifferent.

 Why would administrators be silent or even hostile, considering the impact bullying has on patient care and professional retention? There is little evidence in the literature about the perceptions of nurse administrators toward workplace bullying and if they understand the impact bullying has on patient outcomes and professional retention. This knowledge gap is an urgent area for further investigation. Hoel et al. found that leadership styles predict workplace bullying, both self-reported and observed [47]. Management that is unpredictable or unfair is the strongest predictor, while passive or laissez-faire leadership styles of management, described by our respondents as “doing nothing,” is potentially destructive in itself. Hutchinson and others found that there are five aspects of bullying as organizational corruption; silence and censorship were among them [48]. These authors also identified the use of self-protection tactics by administrators. Jackson emphasizes that without effective management of reports of wrongdoing, internal whistle blowing will continue to have harmful consequences, professionally and personally [49].

The effects of workplace bullying on the quality and safety of patient outcomes are threads that weave throughout the study. The respondents not only reported frustration when administrators did not take action to correct unsafe patient care situations but they also detailed the steps they took to bring attention to these harmful circumstances. When the focus is only on personalities, interpersonal or administrative communications, we lose the significance of the resulting outcomes—they are errors, adverse events, and hazards and need to be investigated as such using a systems change [50].

All employees are responsible for fostering a moral work environment where ethical values are explicit, shared, and guide action [51, 52]. Bullying in the workplace runs counter to the espoused ethical values of health care organizations and must be challenged by managers and front-line nurses who we have been called “upstanders” in this study. To do less than this perpetuates the culture of mistreatment, as confirmed in this study and others [53].

This qualitative study allowed us to focus on the gestalt of nurse bullying in the workplace. We realized that qualitative methods have the potential to guide us to new interventions in a way that quantitative studies cannot. Qualitative inquiry reveals “the complexity, depth and range of living situations relevant to more humanized forms of care” [54].

Todres and colleagues articulate a value framework for humanizing healthcare [54]. Its dimensions express the fundamental elements of humanization specifically related to caring: insiderness, agency, uniqueness, togetherness, sense-making, personal journey, and a sense of place. While much of their work relates to patient care, it is reasonable to apply these same constructs to caregivers and their relationships to each other. Each of the dimensions sits on a continuum from positive to negative. We recognized these humanizing dimensions, or their negative counterparts, in the narratives of our respondents. The nurses' stories gave us an opportunity to be insiders and observe their uniqueness. They searched for meaning and looked for opportunities to make sense of events in their professional lives. Their narratives moved through time, connecting their past and future, creating significant personal journeys.

We propose a number of strategies synthesized from this study for leaders, managers, and staff nurses to use to tackle workplace bullying as follows.

1. All nurses have the responsibility to engage in a process of making things right when faced with workplace bullying.
2. Nurse Leaders must ensure their actions are congruent with the values of the health care organization to build supportive and respectful work environments.
3. Nurse Leaders must work with front-line nurses to discuss the challenges, triggers, and possible solutions to workplace bullying.
4. Nurses should build personal and professional capacity to transform a bystander to upstander when bullying and other aggressive tactics are perpetrated in the workplace.
5. Nurse Leaders must listen to and cocreate a strategic plan with front-line nurses to implement the knowledge from this study in local workplaces to ensure the delivery of quality health care for patients.

While findings from this study have advanced the understanding of workplace bullying in healthcare settings, there are some limitations to be noted. This study was designed to obtain a deeper understanding and generate a grounded theory of nurses' experiences with workplace bullying in the US health care system. While there is much written about the phenomenon of bullying in nursing, literature addressing specific causal relationships, predictive models, or interventions is very limited and therefore was not included in our review of the literature. Clearly this is an area to address in the future. In addition, there is very little written about the experiential nature of bullying, in nursing or other fields; it was crucial to look at the nurses' narratives as their own stories of bullying. We also recognize that one nurse's idea of bullying may not be shared by others. Our participants responded to an online survey, in which an open-ended question asked them to describe a bullying experience. We used the word bullying in the open-ended question and provided a definition, but individual experiences and understandings of the concept may have altered their narratives. Given the virtual nature of the research environment, only respondents with Internet access could participate which could be considered a limitation. In addition, the very anonymity offered to respondents through online submission of narratives may limit our knowledge of them or the circumstances of writing their narratives. On many occasions we found ourselves wishing we knew more about our participants, and because the narrative was not the result of an interaction, either face-to-face or synchronous online discussion, we could not follow up or ask for clarifications.

## 5. Conclusions

Nurses across the United States wrote about their experiences with bullies and bullying in the health care system. They were new and seasoned nurses, from all educational levels, caring for patients in a variety of settings. We asked the participants to describe a bullying situation, and they responded with detailed narratives.

 When nurses were confronted with workplace bullying, they engaged in a process of *making things right*, they placed bullying in context, assessed the situation, took action, and judged the outcomes of their actions. The respondents in this study did not hesitate to acknowledge their own shortcomings, and they were willing to venture their own “theories” as to the motivations of others.

 While there is much discussion in the literature about what constitutes bullying, it is apparent that the nurses in our study recognized the critical elements of the phenomenon. While they understood the emotional consequences of bullying, they were also well aware of how bullying puts patients at risk. Although there has not been any causal relationship established between bullying and patient safety, there is evidence supporting the occurrence of the physiologic and psychological effects of bullying and how they effect wellness, attentiveness, and absenteeism in the workplace. As our respondents noted in their narratives, it is reasonable to conclude that bullying is related in some way to the intersection of professional engagement and the risk for breeches in patient safety, quality of care rendered, and patient outcomes [8, 55]. Understanding the process of *making things right *and using qualitative methods to explore this phenomenon in the future can lead to new strategies and interventions for nurses confronting workplace bullying. And finally, we can extend our hands as collaborators to build effective strategies and successful outcomes.

## Figures and Tables

**Table 1:** Categories and subcategories for *making things right* (Section 3.1).

Core category: making things right (Section 3.2)	
Categories	Subcategories
(1) Placing bullying events (Section 3.3)	(i) Being the newbie
	(ii) Bearing witness
	(iii) In the bull's eye
	(iv) Nurse interrupted
	(v) Odd nurse out
	(vi) In the penalty box
(2) Assessing the situation (Section 3.4)	(i) Reflecting on self
	(ii) Deconstructing the milieu
(3) Taking action (Section 3.5)	(i) Giving/getting support.
	(ii) Speaking up
	(iii) Moving out of the toxic environment
(4) Judging outcomes (Section 3.6)	(i) Constructive—positive
	(ii) Being ignored.
	(iii) Destructive—negative

## References

[1] The New York Times The hospital tyrants and their victims, nurses. http://query.nytimes.com/gst/abstract.html?res=FB0712FF3F5A15738DDDAB0A94D0405B898CF1D3&scp=1&sq=hospital%20tyrants%20and%20their%20vicitms&st=cse.

[2] Baker and McKenzie Worldwide Guide to Termination, Employment Discrimination, and Workplace Harassment Laws. http://digitalcommons.ilr.cornell.edu/lawfirms/50.

[3] Aquino K, Thau S (2009). Workplace victimization: aggression from the target’s perspective. *Annual Review of Psychology*.

[4] Johnson SL (2009). International perspectives on workplace bullying among nurses: a review. *International Nursing Review*.

[5] Farrell GA, Bobrowski C, Bobrowski P (2006). Scoping workplace aggression in nursing: findings from an Australian study. *Journal of Advanced Nursing*.

[6] Lutgen-Sandvik P, Tracy SJ, Alberts JK (2007). Burned by bullying in the American workplace: prevalence, perception, degree and impact. *Journal of Management Studies*.

[7] McKenna BG, Smith NA, Poole SJ, Coverdale JH (2003). Horizontal violence: experiences of Registered Nurses in their first year of practice. *Journal of Advanced Nursing*.

[8] Vessey JA, DeMarco RF, Gaffney DA, Budin WC (2009). Bullying of nurses in the workplace: a preliminary study for developing personal and organizational strategies for the transformation of hostile to healthy workplace environments. *Journal of Professional Nursing*.

[9] Moayed FA, Daraiseh N, Shell R, Salem S (2006). Workplace bullying: a systematic review of risk factors and outcomes. *Theoretical Issues in Ergonomics Science*.

[10] Beecroft PC, Dorey F, Wenten M (2008). Turnover intention in new graduate nurses: a multivariate analysis. *Journal of Advanced Nursing*.

[11] Hegney D, Eley R, Plank A, Buikstra E, Parker V (2006). Workplace violence in Queensland, Australia: the results of a comparative study. *International journal of nursing practice*.

[12] Randle J (2003). Bullying in the nursing profession. *Journal of Advanced Nursing*.

[13] MacIntosh J, O'Donnell S, Wuest J, Merritt-Gray M (2011). How workplace bullying changes how women promote their health. *International Journal of Workplace Health Management*.

[14] Sofield L, Salmond SW (2003). Workplace violence. A focus on verbal abuse and intent to leave the organization. *Orthopaedic Nursing*.

[15] Einarsen S, Hoel H, Zapf D, Cooper CL, Einarsen S, Hoel H, Zapf D, Cooper CL (2011). The concept of bullying at work: the European tradition. *Bullying and Harassment in the Workplace: Developments in Theory, Research, and Practice*.

[16] Monks CP, Smith PK, Naylor P, Barter C, Ireland JL, Coyne I (2009). Bullying in different contexts: commonalities, differences and the role of theory. *Aggression and Violent Behavior*.

[17] Keashly L, Jagatic K, Einarsen S, Hoel H, Zapf D, Cooper CL (2011). North American perspectives on hostile behaviors and bullying at work. *Bullying and Harassment in the Workplace: Developments in Theory, Research, and Practice*.

[18] Leymann H (1990). Mobbing and psychological terror at workplaces. *Violence and Victims*.

[19] Gilmour D, Hamlin L (2003). Bullying and harassment in perioperative settings. *British Journal of Perioperative Nursing*.

[20] Saunders P, Huynh A, Goodman-Delahunty J (2007). Defining workplace bullying behaviour professional lay definitions of workplace bullying. *International Journal of Law and Psychiatry*.

[21] Zapf D, Einarsen S, Hoel H, Vartia M, Einarsen S, Hoel H, Zapf D, Cooper CL (2011). Empirical findings on prevalence and risk groups of bullying in the workplace. *Bullying and Harassment in the Workplace: Developments in Theory, Research, and Practice*.

[22] Dellasega CA (2009). Bullying among nurses. *American Journal of Nursing*.

[23] Monks CP, Coyne I (2011). *Bullying in Different Contexts*.

[24] Owoyemi A, Sheehan M (2011). Exploring workplace bullying in an emergency service organization in the UK. *International Journal of Business and Management*.

[25] Charmaz K, Denzin NK, Lincoln Y (2000). Grounded Theory: objectivist and constructivist methods. *Handbook of Qualitative Research*.

[26] Charmaz K (2006). *Constructing Grounded Theory: A Practical Guide Through Qualitative Analysis*.

[27] Gubrium J, Holstein J, Holstein J, Gubrium J (2008). The constructionist mosaic. *Handbook of Constructionist Research*.

[28] Charmaz K, Holstein J, Gubrium J (2008). The Grounded Theory method. *Handbook of Constructionist Research*.

[29] Markham AN (2006). Ethic as method, method as ethic: a case for reflexivity in qualitative ICT research. *Journal of Information Ethics*.

[30] Kendall L, Johns MD, Chen SLS, Hall GJ (2004). Participants and observers in online ethnography: five stories about identity. *Online Social Research: Methods, Issues, & Ethics*.

[31] Illingworth N (2006). Content, context, reflexivity and the qualitative research encounter: telling stories in the virtual realm. *Sociological Research Online*.

[32] SurveyMonkey http://surveymonkey.com/.

[33] Vessey J, DeMarco R (2007). Bullying scenarios and solutions,. *Nursing Spectrum*.

[34] U.S. Equal Employment Opportunity Commission Prohibited Employment Policies and Practices, Harassment. http://www.eeoc.gov/laws/practices/harassment.cfm.

[35] Glaser BG, Strauss AL (1967). *The Discovery of Grounded Theory: Strategies for Qualitative Research*.

[36] Chen HY, Boore JRP (2009). Using a synthesised technique for grounded theory in nursing research. *Journal of Clinical Nursing*.

[37] Aquino K, Lamertz K (2004). A relational model of workplace victimization: social roles and patterns of victimization in dyadic relationships. *Journal of Applied Psychology*.

[38] Einarsen S, Skogstad A (1996). Bullying at work: epidemiological findings in public and private organization. *European Journal of Work and Organizational Psychology*.

[39] Tepper B (2007). Abusive supervision in work organizations: review, synthesis, and research agenda. *Journal of Management*.

[40] Skogstad A, Matthiesen SB, Einarsen S (2007). Organizational changes: a precursor of bullying at work. *International Journal of Organization Theory and Behavior*.

[41] Archer J, Coyne SM (2005). An integrated review of indirect, relational, and social aggression. *Personality and Social Psychology Review*.

[42] Galen BR, Underwood MK (1997). A developmental investigation of social aggression among children. *Developmental Psychology*.

[43] Underwood MK (2003). *Social Aggression among Girls*.

[44] Lazarus RS, Folkman S (1984). *Stress, Appraisal, and Coping*.

[45] Power S http://www.swarthmore.edu/news/commencement/power.html.

[46] Power S The Seventh Portsmouth Peace Treaty Forum with Samantha Power. http://portsmouthpeacetreaty.org/powerforum2.cfm.

[47] Hoel H, Sheehan,  MJ, Cooper CL, Einarsen S, Einarsen S, Hoel H, Zapf D, Cooper CL (2011). Organisational effects of workplace bullying. *Bullying and Harassment in the Workplace: Developments in Theory, Research, and Practice*.

[48] Hutchinson M, Vickers MH, Wilkes L, Jackson D (2009). “The worse you behave, the more you seem, to be rewarded”: bullying in nursing as organizational corruption. *Employee Responsibilities and Rights Journal*.

[49] Jackson D (2008). Editorial: what becomes of the whistleblowers?. *Journal of Clinical Nursing*.

[50] Reason J (2000). Human error: models and management. *British Medical Journal*.

[51] DeMarco RF (1998). Caring to confront in the workplace: an ethical perspective for nurses. *Nursing Outlook*.

[52] Rodney P, Street A, Storch J, Rodney P, Starzomski R (2004). The moral climate of nursing practice: inquiry and action. *Toward a Moral Horizon: Nursing Ethics for Leadership and Practice*.

[53] Simons SR, Mawn B (2010). Bullying in the workplace–a qualitative study of newly licensed registered nurses. *AAOHN Journal: Official Journal of the American Association of Occupational Health Nurses*.

[54] Todres L, Galvin KT, Holloway I (2009). The humanization of healthcare: a value framework for qualitative research. *International Journal of Qualitative Studies on Health and Well-being*.

[55] Vessey JA, Demarco R, DiFazio R (2010). Bullying, harassment, and horizontal violence in the nursing workforce: the state of the science. *Annual review of nursing research*.

